# ADP-ribosylation from molecular mechanisms to therapeutic implications

**DOI:** 10.1016/j.cell.2023.08.030

**Published:** 2023-10-12

**Authors:** Marcin J. Suskiewicz, Evgeniia Prokhorova, Johannes G.M. Rack, Ivan Ahel

**Affiliations:** 1 Centre de Biophysique Moléculaire, CNRS, Orléans, France; 2 Sir William Dunn School of Pathology, University of Oxford, Oxford, UK; 3 MRC Centre of Medical Mycology, University of Exeter, Exeter, UK

## Abstract

ADP-ribosylation is a ubiquitous modification of biomolecules, including proteins and nucleic acids, that regulates various cellular functions in all kingdoms of life. The recent emergence of new technologies to study ADP-ribosylation has reshaped our understanding of the molecular mechanisms that govern the establishment, removal, and recognition of this modification, as well as its impact on cellular and organismal function. These advances have also revealed the intricate involvement of ADP-ribosylation in human physiology and pathology and the enormous potential that their manipulation holds for therapy. In this review, we present the state-of-the-art findings covering the work in structural biology, biochemistry, cell biology, and clinical aspects of ADP-ribosylation.

## Teaser

ADP-ribosylation of proteins and nucleic acids regulates various cellular functions in all kingdoms of life, and its manipulation holds potential for human therapeutics. 60 years after the discovery of ADP-ribosylation, this review provides state-of-the-art insights on structural biology, biochemistry, cell biology, and clinical aspects.

## Introduction

The polymerase activity capable of producing a biopolymer from nicotinamide adenine dinucleotide (NAD^+^) was first identified in the 1960s,^1^ during the heyday of research into canonical DNA and RNA polymerases to which it appeared analogous. In this process—now known as ADP-ribosylation—NAD^+^ is split into ADP-ribose and nicotinamide (NAM), and ADP-ribose is added to an acceptor (typically an amino-acid residue on a protein) or to another ADP-ribose unit in a growing poly(ADP-ribose) (PAR) chain. In hindsight, the originally observed activity was mainly due to poly(ADP-ribosyl) polymerase 1 (PARP1), the major ADP-ribosylation enzyme in human cells. Soon after, researchers working on the causative agent of diphtheria found that the toxin produced by these bacteria kills host cells by transferring a single ADP-ribose moiety from NAD^+^ onto an essential cellular protein.^2^ Thus, ADP-ribosylation was discovered almost simultaneously as a eukaryotic and a prokaryotic phenomenon.

Today, we know that ADP-ribosylation-based signaling systems are widespread in all kingdoms of life and function both in virulence and immunity and as a regulatory mechanism, primarily by controlling intramolecular interactions. The last two decades have seen the maturation of the field, which has broadened its focus from PARP1 and bacterial toxins to various PARP proteins and other ADP-ribosylation “writers,” “erasers,” and “readers.” ADP-ribosylation also proved its therapeutic relevance, particularly with the two seminal studies in 2005 that reported synthetic lethality between inhibition of PARP1 or PARP2 and *BRCA* (breast cancer gene) deficiency,^3^^,^^4^ paving the way for the current widespread use of PARP inhibitors (PARPi) in treating specific cancer types.

In the last several years, ADP-ribosylation has experienced a renewed surge of interest with the discovery of novel phenomena such as nucleic acid-linked ADP-ribosylation, the regulation of PARP1 by an accessory factor, HPF1 (histone PARylation factor 1), PAR’s role as a trigger of macromolecular condensate formation, and the close interplay between ADP-ribosylation and ubiquitylation, to name just a few. The recent period has also seen the development of new small-molecule inhibitors—including those against PARPs other than PARP1 or PARP2—and other dedicated research tools such as antibody-like reagents and chemical probes.^5^ Moreover, the field has greatly accelerated due to technological advancements in proteomics, cryo-electron microscopy, and computational protein structure prediction, with the next decade promising to yield further breakthroughs in our understanding of ADP-ribosylation signaling and its therapeutic implications.

This review aims to present a comprehensive overview of the fast-developing ADP-ribosylation field with a focus on the fundamental concepts on the one hand and the recent research advances on the other. We follow the increasing complexity at which biological phenomena can be described and further explored, from the atomic scale (chemistry of ADP-ribosylation) through the molecular scale (structure and mechanism of action of ADP-ribosylation writers, erasers, and readers) to the cellular and organismic scale (biological role of ADP-ribosylation in eukaryotes, prokaryotes, and viruses). We go back and forth between these levels to highlight how they are interdependent and how this is integrated with an evolutionary perspective. In the final sections, we discuss the therapeutic relevance of ADP-ribosylation, leading to an outlook of urgent remaining questions concerning both the fundamental understanding and therapeutic potential of ADP-ribosylation.

## Chemical and molecular principles of ADP-ribosylation

**Figure 1:**
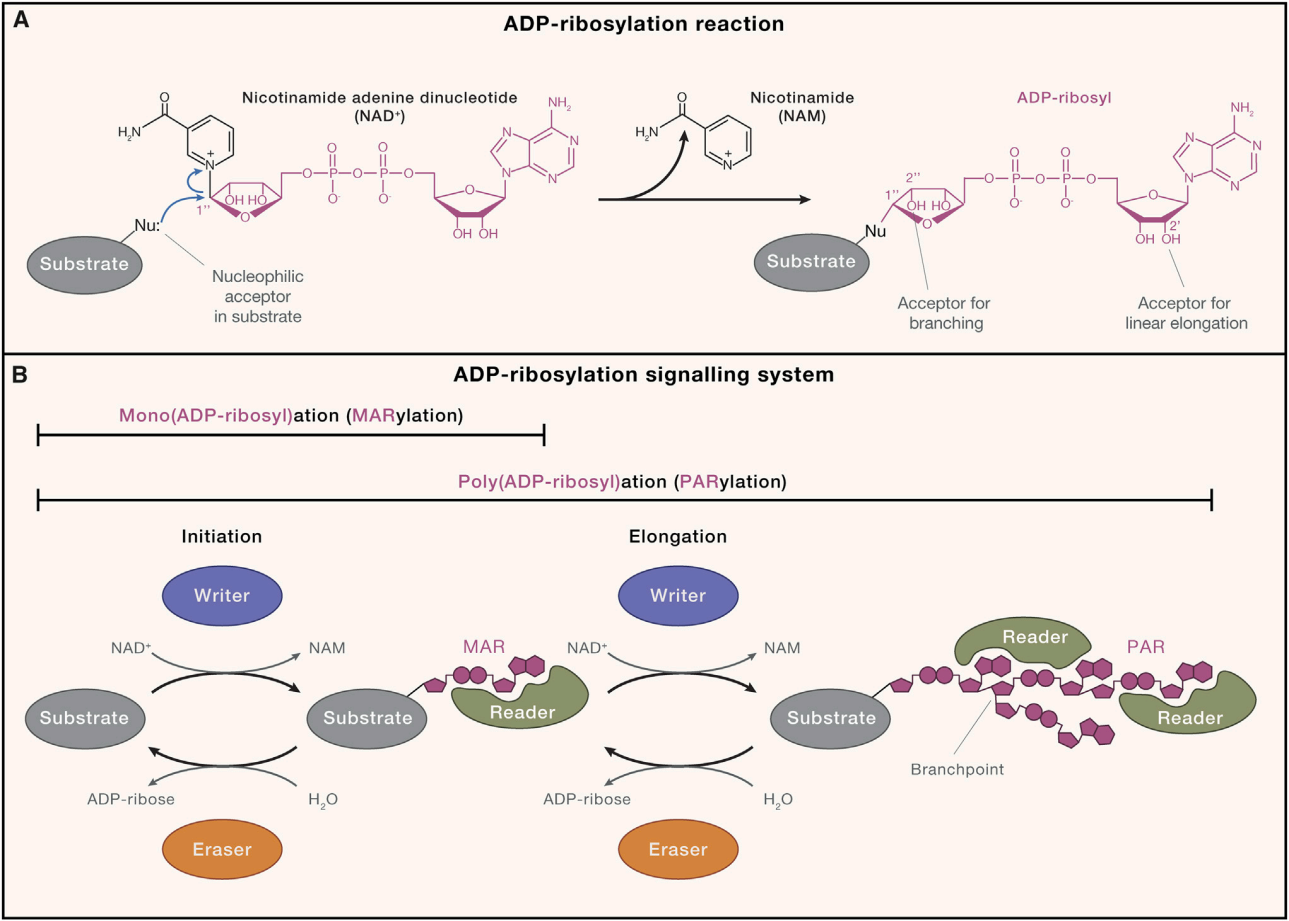
The ADP-ribosylation reaction (A) Simplified mechanism of the ADP-ribosylation reaction. Nucleophilic attack of a suitable nucleophilic acceptor group (Nu) in a substrate on the C1″ atom of NAD^+^ is indicated with the blue curly arrow. Following the addition of the initial ADP-ribose moiety to the substrate, the ADP-ribosylation reaction can be repeated with either the 2′ or 2″ hydroxyl group of the initial ADP-ribose serving as a nucleophilic acceptor for linear or branched chain elongation, respectively. (B) Regulation of ADP-ribosylation by writers and erasers and its recognition by readers. The ADP-ribosylation reaction that stops at the initiation stage produces mono(ADP-ribosyl)ation or MARylation, while the initiation followed by repeated rounds of elongation generates poly(ADP-ribosyl)ation (PARylation). Initiation and elongation stages can be associated with distinct sets of writers, erasers, and readers.

In basic molecular terms, ADP-ribosylation is an enzymatic modification reaction that proceeds via the transfer of the ADP-ribose moiety from NAD^+^ as a donor onto an acceptor group within a substrate^6^ (Figure 1A). ADP-ribose becomes linked to the acceptor via the anomeric C1″ atom. The possible ADP-ribosylation substrates include proteins—making ADP-ribosylation a type of protein post-translational modification (PTM)—but also other biomolecules, particularly nucleic acids. By regulating interactions, localization, and half-life of molecules and orchestrating condensation events, ADP-ribosylation regulates many different cellular processes, as discussed in more detail in later sections.

### Writers, readers, erasers, and feeders

The regulatory role of ADP-ribosylation necessitates its timely and specific establishment and removal, as well as its physiologically appropriate interpretation in terms of downstream events. ADP-ribosylation-based regulation, therefore, depends on writers (enzymes that establish the modification), erasers (enzymes that remove it), and readers (proteins or domains that bind to it, which can also be a part of signal interpretation). Recently, a category of “feeders” has been proposed to describe enzymes—particularly the nicotinamide-nucleotide adenylyltransferases (NMNATs) family proteins—that replenish NAD^+^ from NAM, in some contexts serving as a necessary component of an ADP-ribosylation pathway.^7^ Different NMNATs control the NAD^+^ concentration in specific cellular compartments.^8^

### Acceptors

Protein ADP-ribosylation can be attached to various acceptor amino-acid residues, such as serine, threonine, tyrosine, glutamate, aspartate, cysteine, arginine, lysine, and histidine, via *O*-, *S*-, or *N*-glycosidic linkages.^6^^,^^9^ Attachment to amino acids characterized by different chemical structures and properties creates potentially orthogonal ADP-ribosylation pathways that rely on distinct writers, erasers, and—possibly—readers, thus having a potential to simultaneously influence different aspects of cellular physiology in a specific manner. For nucleic acid substrates, ADP-ribosylation of terminal phosphates and bases has been reported.^10^^,^^11^^,^^12^^,^^13^^,^^14^^,^^15^^,^^16^

### MARylation, PARylation, and the “PAR code”

Once a single mono-ADP-ribose (MAR) moiety is attached to a substrate (mono(ADP-ribosyl)ation or MARylation), it can either serve as a regulatory signal in its own right or be elongated into a PAR chain (Figure 1B). PAR synthesis (PARylation), which can be catalyzed by some ADP-ribosylation writers, including the best-studied PARP1, proceeds via the attachment of further ADP-ribose moieties to the initial modification. There is emerging evidence that initiation and elongation steps can be regulated separately at the level of both synthesis and removal.^17^ The dominant attachment point for new ADP-ribose moieties in a growing PAR chain is the 2′ hydroxyl group on the last-added ADP-ribose, but chains can also branch out through ADP-ribose attachment to the 2′' hydroxyl group (Figure 1A). Differences in PAR chain length and branching frequency have a potential to translate into differential resistance to hydrolysis or preference for distinct sets of readers, presumably giving rise to a “PAR code.”^18^ A specific ubiquitin E3 ligase class, DELTEX, has recently been found to be able to add ubiquitin to the 3′ hydroxyl of the ADP-ribose molecule linked to a protein,^19^ producing a hybrid ADP-ribose-ubiquitin chain of so far unclear cellular relevance and demonstrating an example of additional ways in which the ADP-ribosylation signals could be remodeled.

## Emergence and evolution of ADP-ribosylation

The evolutionary emergence of ADP-ribosylation, like that of other key modifications, was likely driven by the ready availability of the required donor molecule, in this case, NAD^+^, in all cells due to its fundamental metabolic function. Moreover, NAD^+^ is intrinsically an efficient donor due to the fact that the NAM moiety is a good leaving group, i.e., has a propensity for accepting electrons and detaching from NAD^+^. Because NAD^+^ was abundant and reactive, enzymes that accelerate its reaction with acceptors could gradually emerge, and the simultaneous role of NAD^+^ as a primary metabolite enabled an advantageous coupling between metabolism and regulatory mechanisms. In addition, ADP-ribosylation, with its bulky size and highly negative charge, was arguably a particularly attractive mechanism for evolution to harness for blocking existing interactions and initiating new, specific contacts with ADP-ribosylation readers.

In terms of the evolutionary timeline, modification reactions, including ADP-ribosylation, are thought to have emerged in bacteria as part of the rapid expansion of secondary metabolism and conflict- and immunity-related systems during the great oxygenation event.^20^ This period, which took place around 2 billion years ago, is an important time point in Earth’s history, characterized by both the extinction and the emergence of various species, coinciding with the rise in oxygen levels. For ADP-ribosylation, the connection with conflict and immunity is particularly well-established and is still visible in the form of numerous extant ADP-ribosylation-based exotoxins, toxin-antitoxin modules, as well as phage defense and antibiotic-modifying systems.^21^ The main ADP-ribosylation writer domain—the (ADP-ribosyl)transferase (ART) domain—likely emerged in this context, as did ADP-ribosylation reader and eraser domains such as the macrodomain.^20^ The ART domains and macrodomains, in particular, show high divergence on the primary and even tertiary structure level, with frequently observed extensions and modifications of the core structures described below. In fact, the homology between PARP1 and bacterial ADP-ribosylating toxins, both of which contain an ART domain, had not been evident until relevant crystal structures were solved, revealing structural similarity,^22^^,^^23^^,^^24^ and the same was the case for PAR glycohydrolase’s (PARG’s) relatedness to macrodomain enzymes.^25^ In eukaryotes, ADP-ribosylation not only kept the conflict and immunity dimension but also became utilized for internal regulatory processes such as DNA repair. The ancestral *PARP* gene in eukaryotes dates back to before the last eukaryotic common ancestor.^26^ ARTs have been acquired by eukaryotes several times through horizontal gene transfer. Below, we begin a more detailed discussion of ADP-ribosylation by describing, in turn, ADP-ribosylation writers, erasers, and readers, focusing on their structural and mechanistic aspects.

## ADP-ribosylation writers

The key aspect of all enzymatic ADP-ribosylation signaling systems is the writing reaction, whereby ADP-ribose becomes transferred from NAD^+^ onto substrates and, in some cases, polymerized into PAR chains. Below, we review what is known about various writer enzymes, primarily from a mechanistic and structural perspective and with a focus on human enzymes.

### Writer enzymes

#### The ART domain and its ARTC and ARTD subtypes

**Figure 2:**
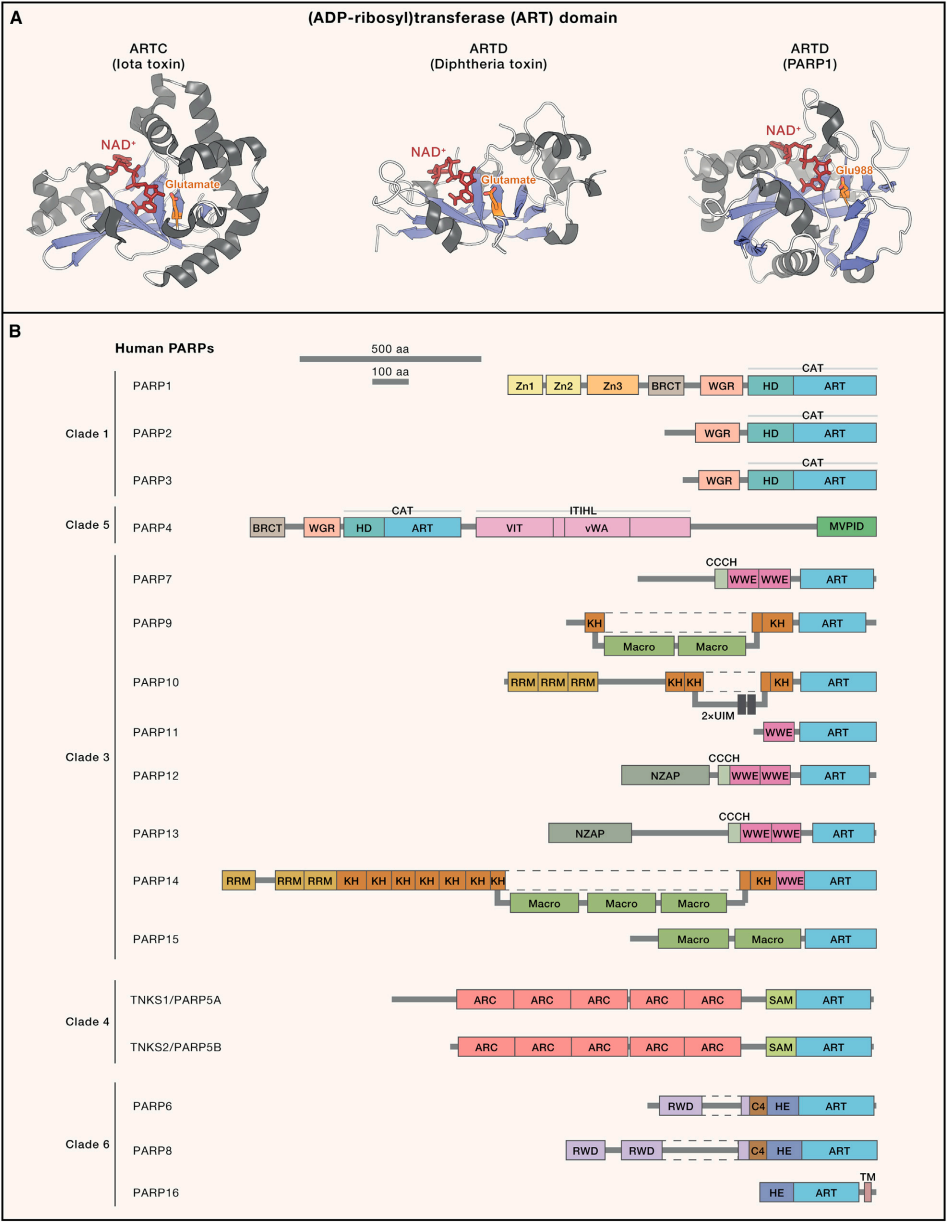
(ADP-ribosyl)transferases (ARTs) and domain organization of PARPs (A) Examples of the ART domain from cholera and diphtheria toxin-like families (ARTC and ARTD, respectively) are provided showing conserved features (a central β sheet, a conserved catalytic glutamate residue, a similar conformation of the bound NAD^+^ donor) as well as structural divergence. PDB entries used for the figure, PDB: 1XTC,^23^ 1TOX,^24^ and 6BHV.^28^ (B) Domain composition of all human PARP proteins based on both experimental studies and computational predictions, including AlphaFold 2 models. PARPs are grouped into evolutionary clades. Parts of “split” domains that are consecutive in structure but not in sequence are connected with a dashed line. Zn1–3, zinc-finger domains 1–3; BRCT, BRCA1 C terminus; WGR, tryptophane-glycine-arginine; HD, helical domain; ART, (ADP-ribosyl)transferase; CAT, catalytic domain; VIT, vault protein inter-alpha-trypsin; vWA, von Willebrand factor type A; ITIHL, inter-alpha-trypsin heavy chain-like; MVPID, MVP-interacting domain; CCCH, cysteine-cysteine-cysteine-histidine zinc finger; WWE, tryptophane-tryptophane-glutamate; KH, ribonucleoprotein K homology; RRM, RNA-recognition motif; UIM, ubiquitin-interacting motif; NZAP, N-terminal domain of zinc-finger antiviral protein; ARC, ankyrin repeat cluster; SAM, sterile-alpha motif; RWD, RING-fingers, WD proteins, and DEXDc-like helicases; C4, four-cysteine zinc finger; HE, helical extension; TM, transmembrane helix.

The main domain responsible for ADP-ribosylation in both pro- and eukaryotes is the ART domain, which has a characteristic core with a “split β sheet,” composed of two three-stranded antiparallel sheets and two helical regions (Figure 2A). Here, we refer to proteins that contain the ART domain as ART proteins. Two main branches of the ART superfamily, the ARTD family (which includes PARPs) and the ARTC family, are named after paradigmatic bacterial diphtheria and cholera toxins and are sometimes defined by triads of partially conserved active-site residues, H-Y-E and R-S-E, respectively. However, the distinction is only partly useful, as some proteins that clearly belong to the ART superfamily are distantly related to both paradigmatic bacterial toxins. In human cells, we find a few such diverged ARTs, of which NEURL4 is most studied,^20^^,^^27^ in addition to PARP enzymes of the ARTD family and several ARTCs. This rich repertoire of verified and potential ADP-ribosylation writers still awaits full characterization.

#### PARP proteins

The PARP group of the ARTD family includes 17 canonical members in humans that share a C-terminal “PARP-type” ART domain and otherwise vary in domain composition^6^^,^^29^ (Figure 2B). PARPs, which have been subdivided into clades according to their evolutionary relationships, are found primarily in metazoan and less frequently in bacterial and non-metazoans eukaryotes.^26^ The acronym “PARP”—initially indicating a polymerase activity—is used to refer to all family members, regardless of whether or not they possess PARylation or indeed any detectable ADP-ribosylation activity.^6^ In fact, the catalytic activity of the majority of PARPs, like those of bacterial toxins and human ARTCs, is likely limited to the MARylation reaction.^6^^,^^9^ The best-studied ARTs that can catalyze PARylation are DNA repair-associated PARP1 and PARP2 and tankyrases TNKS1 and TNKS2 (previously also called PARP5A and PARP5B). Some PARPs might be pseudoenzymes that lost catalytic activity,^9^ but this is difficult to establish with certainty because ARTs might be specialized for particular substrates or require auxiliary factors for physiological activity. Overall, the diversity in terms of domain composition, activity, substrate specificity, and, as will be discussed next, localization, likely reflects the expansion of PARPs to fulfill a broad range of cellular functions.

#### Localization of human ART proteins

PARPs are primarily localized to the nucleus or cytosol, including the perinuclear area, with some of them shuttling between these locations. Multiple PARPs, including TNKS1, PARP12, PARP13, PARP14, and PARP15, can localize to stress granules, cytoplasmic condensates of proteins, and RNA that are formed in response to various stresses.^30^ PARP4 associates with vaults, enigmatic ribonocluoprotein particles present in the cytoplasm.^31^ TNKS1 and TNKS2 are seemingly unique among PARPs in existing, independently of ADP-ribosylation-induced clustering, as noncovalent protein polymers, which are formed via their sterile-alpha motif (SAM) domains. These filaments contribute to a punctate localization and have an impact on functional properties.^32^^,^^33^ The only human PARP that is known to be membrane-associated is PARP16, which is linked with the endoplasmic reticulum (ER) membrane or nuclear envelope through a transmembrane helix, with the ART domain and most of the protein positioned on the cytoplasmic side.^34^^,^^35^ By contrast, human ARTCs (also known as ARTs), which can be either membrane-anchored through a glycosylphosphatidylinositol modification (ARTC1–4) or secreted (ARTC5), have their ART domains positioned in the ER lumen or outside the cell.^36^ The PARP-like protein NEURL4 has recently been reported to function in the mitochondria.^37^ This complex picture, which is likely far from comprehensive, hints at different PARPs occupying diverse, partially overlapping, spatial niches. This might in turn reflect functional compartmentalization, although there might also be functional crosstalk between different PARPs, especially when they are situated in the same locations.

#### Noncanonical ADP-ribosylation writers

A special case of an ART family member is represented by TRPT1 (Tpt1 in yeast, KptA in *Escherichia coli*), a conserved enzyme related to PARPs, which uses NAD^+^ to dephosphorylate RNA as its main function,^38^ but can also ADP-ribosylate RNA on terminal phosphates,^10^^,^^11^ similarly to several human PARPs.^10^^,^^11^^,^^12^^,^^13^ In addition to ARTs, some members of the sirtuin family (including human SIRT4, SIRT6, and SIRT7)—which primarily work as NAD^+^-dependent deacylases—have been reported to possess a protein ADP-ribosylation activity,^39^^,^^40^^,^^41^ which remains to be better characterized. In contrast to human sirtuins, where this activity might be secondary, M-class sirtuins found in bacterial and fungal pathogens appear to be primarily protein ADP-ribosylating enzymes.^42^

### Mechanism of catalysis and substrate binding by ARTs

Chemically speaking, ADP-ribosylation is a nucleophilic substitution reaction whereby an acceptor attacks the anomeric C1″ atom of NAD^+^ while NAM departs as the leaving group. This results in a covalent acceptor-ADP-ribose linkage and an inversion of stereochemistry at the C1″ position (i.e., from β to α). It has been debated whether NAM departure precedes or occurs simultaneously with acceptor modification (S_N_1 [nucleophilic substitution type 1, unimolecular]/dissociative vs. S_N_2 [nucleophilic substitution type 2, bimolecular]/associative mechanism, respectively), and a hybrid of these extremes is likely, with the leaving group being almost departed when the bond to the acceptor begins to be formed.^43^^,^^44^ Generally speaking, the catalytic potential of ARTs relies on their ability to activate NAD^+^, recruit the substrate, and activate the ADP-ribosylation acceptor. Some ARTs, including PARP1, are highly active, making it challenging to produce fully inactive mutants without losing NAD^+^ binding or protein stability. Other ARTs appear lowly active, possibly because their correct substrates or necessary cofactors have not yet been identified. In the absence of a good substrate, some ARTs tend to hydrolyze NAD^+^.^45^ ARTs that show high substrate specificity achieve it through a larger-than-typical substrate-binding surface, as seen for DNA-modifying toxins.^15^^,^^46^ In all these cases, however, the chemical details of the ADP-ribosylation reaction—discussed briefly below—are important for understanding the biological output generated by ADP-ribosylation writers.

#### NAD^+^ activation

**Figure 3:**
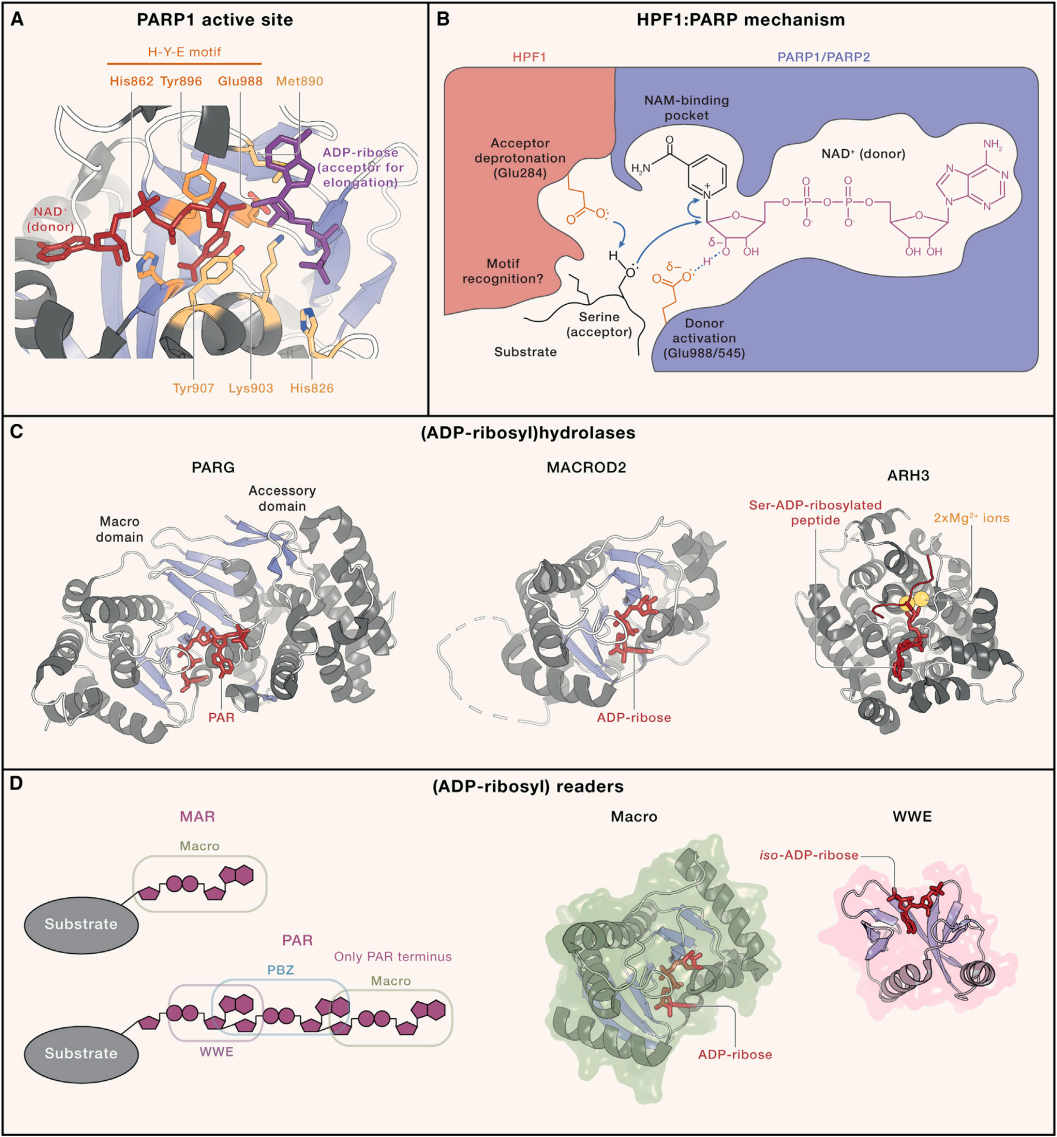
Mechanisms and structures of writers, erasers, and readers of ADP-ribosylation (A) The structural model of the active site of PARP1 during the catalysis of PAR chain elongation. The donor NAD^+^ and a fragment of the acceptor ADP-ribose molecule are shown according to the PDB entries, PDB: 6BHV^28^ and 1A26.^49^ Residues implicated in catalysis as well as donor and acceptor binding are shown. (B) The mechanism of serine ADP-ribosylation catalysis by the composite active site built by PARP1 and HPF1. Possible movement of electrons during the reaction is indicated with blue curly arrows. For figure clarity, NAD^+^ is not shown in correct stereochemistry (see Figure 1A). (C) Structures of (ADP-ribosyl)hydrolases from the macrodomain (PARG and MacroD2) and ARH (ARH3) families. Co-crystallized substrates are shown. PDB entries used for the figure, PDB: 4L2H,^50^ 4IQY,^51^ and 7AKS.^52^ (D) Scheme showing the specificity of various reader domains for their cognate MAR and PAR signals (left) and structures of two representative reader domains co-crystallized with their ligands (right). PDB entries used for the figure, PDB: 2BFQ^53^ and 4QPL.^54^

During the ADP-ribosylation reaction, ribose temporarily takes on an oxocarbenium character (i.e., has a positive charge delocalized between a carbon and an oxygen atom in a ring), which has implications for how ARTs can encourage NAD^+^ reactivity. ARTs use various mechanisms to promote NAM departure and stabilize the oxocarbenium, including binding NAD^+^ in a conformation that favors a stabilizing interaction between orbitals known as orbital hyperconjugation^43^ and using a hydrophobic pocket to desolvate the NAM ring.^43^^,^^47^ Another mechanism involves an interaction between the catalytic glutamate present in many ARTs (the shared E in H-Y-E and R-S-E motifs; E988 in PARP1) (Figures 2A and 3A) and the 2′' ribose hydroxyl, which serves to indirectly counter the positive charge^47^ (Figure 3B). Moreover, ARTs bind NAD^+^ in an atypical conformation (Figure 2A), which is conserved across various ARTs but is stabilized through different residues in ARTD versus ARTC family: histidine and tyrosine from the H-Y-E motif (Figure 3A) and arginine-serine from the R-S-E motifs, respectively.^48^ This conformation could impose strain onto NAD^+^, which can be relieved during the reaction.

#### Acceptor activation

The catalytic glutamate residue has also been proposed to assist in acceptor deprotonation in PARP1 during PAR chain elongation, explaining the MARylation-only activity of PARP1 E988 mutants.^55^ In cases where a negatively charged group (e.g., a protein glutamate residue) is acting as an acceptor of ADP-ribosylation, the substrate does not need deprotonation and itself may take over the role of the catalytic glutamate in stabilizing the oxacarbenium ion.^44^ This could explain some cases where PARPs that lack the catalytic glutamate retain catalytic activity.^9^ An interesting paradigm is represented by serine-targeting complexes composed of PARP1 or PARP2 and the auxiliary factor HPF1.^56^^,^^57^ These complexes have a compound active site with two catalytic glutamates: one (for oxocarbenium stabilization) provided by the PARP enzyme and the other (likely for serine deprotonation) provided by HPF1^58^ (Figure 3B). Generally speaking, the way ARTs recognize and activate specific acceptors is arguably the least understood facet of ADP-ribosylation writing, and new insights in this area based on detailed mechanistic studies are required.

#### Substrate recruitment

Like other enzymes that catalyze modification reactions, ARTs tend to perform a two-step discrimination of substrates.^48^ The first step involves recruitment of a substrate to an ART or their co-recruitment to the same location. Tankyrase substrates appear to be recruited to TNKS1 or TNKS2 via tankyrase-binding motifs (TBMs), which bind to the substrate-binding ankyrin repeat clusters (ARCs) in tankyrases.^59^ PARP1 is co-localized with its substrates through interactions with damaged chromatin or with previously deposited PAR signals.^48^ We discuss the interaction of PARP1 and related PARPs with DNA and chromatin in more detail in the next section, where we explain that DNA binding serves an additional role of allosterically activating these PARPs while they are in proximity of their cognate substrates. The second step of substrate selection consists in the recognition of the modified site and its immediate surrounding. In the case of PARP1 and PARP2, the most-commonly modified K-S motif is recognized by the cleft between PARP and HPF1.^57^^,^^58^ During chain elongation, PARP1 recognizes a protein-linked ADP-ribose moiety as an acceptor, with two alternative binding modes leading to either linear PAR chain elongation or branching^49^ (Figure 1B).

### DNA binding and DNA-dependent activation of DNA repair-associated PARPs

The best-studied ART enzyme, PARP1, and its two closest homologs, PARP2 and PARP3, interact with both damaged and undamaged DNA and chromatin. PARP1, PARP2, and PARP3 associate with DNA breaks using the WGR domain (named after conserved residues), and PARP1 additionally uses three zinc fingers for this task^60^^,^^61^ and can bind to intact DNA via its BRCT (BRCA1 C terminus) domain.^62^ PARP2 has the ability to bridge two blunt double-stranded DNA ends.^63^^,^^64^^,^^65^ Although PARP1 binds to model DNA breaks with high, nanomolar affinity, rapid exchange of PARP1 molecules on DNA is observed both *in vitro* and in cells.^66^^,^^67^ PARP1 has been suggested to travel between DNA segments via a “monkey bar” mechanism whereby association with a new DNA break stimulates the release of PARP1 from a previous break.^68^ *In vitro*, PARP1 strongly binds histones and a chromatin fragment (DNA-wrapped trinucleosomes) that lacks exposed DNA ends.^69^ Furthermore, PARP1 binding has been reported to promote nucleosome assembly^69^ and trigger chromatin condensation.^70^^,^^71^ Whereas binding to histones, nucleosomes, and intact DNA might serve other functions, binding to various types of DNA breaks is known to allosterically activate PARP1, PARP2, and PARP3, allowing them to produce signals when and where DNA damage took place.

The DNA-dependent activation of PARP1, PARP2, and PARP3 is mediated by partial unfolding and displacement of their autoinhibitory helical domain (HD), which otherwise blocks NAD^+^ access, as revealed by recent hydrogen-deuterium exchange and crystallography analyses.^28^^,^^60^^,^^72^^,^^73^ Although PARP1 is activated by various DNA structures, PARP2 and PARP3 require 5′-phosphorylated DNA ends for their activation.^63^^,^^74^ Once activated, PARP1, PARP2, and PARP3 modify themselves and other substrates. PARP1 automodification is mainly localized to the region between the BRCT and WGR domains and especially to three serine residues, which modification depends on HPF1.^75^ PARP1 automodification negatively affects its association with DNA and chromatin, preventing prolonged bulk residence on damaged chromatin (also known as “PARP trapping”).^75^^,^^76^^,^^77^

The above discussion of ADP-ribosylation writers reflects years of research in the field, revealing a broad mechanistic complexity of these enzymes and their diversity despite reliance, in most cases, on a similar catalytic domain. Prior to delving into biological functions of ADP-ribosylation writers, we review structural and mechanistic aspects of two other key players in ADP-ribosylation signaling systems: erasers and readers.

## ADP-ribosylation erasers

The ability to terminate the ADP-ribosylation signal is as important as its establishment in order to ensure a dynamic and tightly regulated signaling process. Therefore, enzymes that catalyze either full or partial reversal of ADP-ribosylation have emerged in the course of evolution. Various aspects of these enzymes have been recently reviewed.^78^ Here, we offer an overview of their structural and mechanistic aspects, including the most recent insights.

### Eraser enzymes

To date, three protein superfamilies have been identified to fully reverse the modification, corresponding to proteins containing the following domains: the macrodomains, (ADP-ribosyl)hydrolase (ARH) domains, and, most recently, NADAR (named after NAD^+^ and ADP-ribose) domains. Both macrodomain and ARH hydrolases have been identified in all kingdoms of life and some viruses.^79^ Various eraser enzymes from these two superfamilies differ in their substrate specificities (PAR vs. MAR, various amino acid-ADP-ribose linkages) and efficiency, which translates into their different biological roles. In addition to full hydrolysis by erasers, the ADP-ribosylation signal can be converted, at least *in vitro*, into a 5-phosphate-ribosyl modification by members of phosphodiesterase families Nudix and ectonucleotide pyrophosphatase/phosphodiesterase (ENPP).^78^^,^^80^

Another aspect that differs between individual ADP-ribosylation erasers is their subcellular localization, which is supple and can dynamically change in response to cellular events. The primary PAR-degrading enzyme, the macrodomain-containing hydrolase PARG, has different isoforms that preferentially localize to the nucleus, cytoplasm, or the mitochondrial matrix, respectively.^81^^,^^82^ Other hydrolases show primarily mitochondrial (MacroD2), cytoplasmic (ARH1), nuclear and cytoplasmic (TARG1, MacroD1), or more broad or variable, e.g., nuclear, cytoplasmic, and mitochondrial (ARH3), localization.^78^ Like for writers, differences in localization might reflect functional compartmentalization of erasers and could indicate functional association between specific erasers and writers.

The fact that ADP-ribosylation erasers can utilize one of three completely different domains makes them more diverse in basic mechanistic terms than ADP-ribosylation writers generally are. For this reason, we divided the discussion below into independent parts dedicated to different domains. Some functional aspects that we briefly addressed for writers—for example mechanisms of allosteric activation or protein substrate recognition—remain relatively unexplored in the case of erasers.

### Structure, mechanism, and specificity of macrodomains

Macrodomains (Figure 3C, left and middle) share a common α/β/α sandwich core motif consisting of a mixed five-stranded β sheet flanked by five α helices.^78^ This core is often extended by additional α helices and β sheets. The ADP-ribose binding cleft in macrodomains is situated on the crest of the β sheet with important contacts provided by other secondary structural elements. The region around the active site has undergone extensive diversification, allowing macrodomains to hydrolyze a range of substrates, including PAR, glutamate/aspartate-linked ADP-ribosylation, and *O*-acetyl-ADP-ribose among others,^51^^,^^78^ as well as acting as a catalytically inactive ADP-ribosylation reader domains. Indeed, macrodomains show enormous sequential diversification, and novel classes keep being identified.^53^

#### PARG-like and MacroD-type macrodomains

In humans, a PARG-like macrodomain (Figure 3C, left) is found in a single protein, PARG, while MacroD-type macrodomains (Figure 3C, middle) can be found in a family of proteins consisting of three members (MacroD1, MacroD2, and GDAP2). The PARG-like and MacroD-type macrodomains show some similarities in substrate coordination and activation.^78^ Both classes catalyze the cleavage of an *O*-glycosidic bond from the C1″ of the distal ribose. The latter is forced into a strained conformation through an aromatic residue and tight coordination of the 2″ and 3″ hydroxyl moieties. The PARG-catalyzed reaction utilizes an S_N_1-type mechanism in which the catalytic EE dyad polarizes the distal ribose. It has been suggested that the microenvironmental changes upon ligand binding lead to an increase of the pK_a_ of a glutamate residue, thus allowing formation of a protonated intermediate that can act as a nucleophile to attack the glycosidic bond. This leads to the formation of an oxocarbenium, which quickly reacts with solvent water to form ADP-ribose as a product. Although MacroD-type macrodomains also polarize the distal ribose using various sub-class specific residue combinations,^83^ they do not contain residues that would allow direct nucleophilic cleavage. Instead, MacroD-type macrodomains employ a substrate-assisted mechanism in which a water molecule is coordinated coplanar to the C1″ and activated by the α-phosphate of the ADP-ribosyl, which allows the reaction to proceed through an S_N_2 type mechanism.^78^^,^^83^^,^^84^^,^^85^

In MacroD-type macrodomains associated with ADP-ribosylating sirtuins in some bacteria, the catalytic loop is exchanged by a zinc-coordinating loop, allowing the enzyme to cleave not only *O*-glycosidic bonds to acidic residues^42^ but also the *S*-glycosidic linkage to cysteine residues.^86^ Although the physiological relevance of this reaction remains elusive, this activity may be a useful tool to study cysteine-linked ADP-ribosylation detected in higher organisms.

#### ALC1-like macrodomains

Although ALC1 itself is an ADP-ribosylation reader (discussed below), some ALC1-like proteins, including TARG1 in humans, are active hydrolases, thus forming the third class of macrodomain erasers. The ALC1-like class differs substantially from the PARG-like and MacroD-types classes. TARG1, similar to MacroD1/MacroD2, catalyzes the hydrolysis of glutamate/aspartate-linked ADP-ribosylation, albeit utilizing a distinct mechanism. The key residues of catalysis are a D-K dyad located on the opposite side of the binding cleft relative to PARG and MacroD1/MacroD2. During the catalysis, the aspartate residue activates the lysine residue, which in turn acts as a nucleophile to form a covalent intermediate with the protein substrate, which later is resolved in a glutamate-dependent manner.^87^ Noteworthy, variations of this mechanism must exist as several ALC1-like macrodomains from other organisms can facilitate this reaction despite lacking the lysine and aspartate residues conserved in mammals.^21^^,^^78^

### Structure, mechanism, and specificity of ARHs

The ARH superfamily of hydrolases fills a vital gap in the ADP-ribosylation eraser landscape by enabling the removal of serine- and arginine-linked modification not catalyzed by human macrodomains. They are mostly α-helical globular proteins (Figure 3C, right), which show overall less structural diversity than macrodomains.^78^ The ADP-ribose binding occurs in a deep central cleft where the distal ribose interacts with the catalytic binuclear metal center. A significant diversification of the ligand coordination is the accommodation of the adenosine moiety, which in ARH1 is coordinated coplanar with the protein surface and shielded from the solvent by an aromatic residue, whereas in ARH3 it is stacked perpendicular to the surface between two aromatic residues.^78^^,^^88^ Despite a high degree of similarity between their metal centers, different ARH classes have evolved different metal preferences. Although the human enzymes most likely utilize magnesium ions for the reaction in cells, they can also accommodate manganese ions, and bacterial enzymes of the DraG-like class were shown to prefer manganese and iron(II) ions.^52^ For ARH3, it has been shown that substrate binding induces several conformational changes, most notably the re-orientation of the flexible glutamate flap (also termed E41-flap in humans), which positions the catalytic glutamate residue for catalysis and leads to a high-energy state of one of the magnesium centers.^52^ The mechanistic details of reversal of arginine ADP-ribosylation by either ARH1 or DraG remain so far elusive.

### NADAR domains’ ADP-ribosylation eraser function

The most recent addition to the list of known ADP-ribosylation eraser superfamilies are NADAR domains, which appear to specifically remove ADP-ribosylation that is attached to single-strand DNA (ssDNA) via the guanine base. This novel guanine demodification activity was recently discovered for the NADAR protein DarG1, which functions as an antitoxin that counteracts the guanine ADP-ribosylating toxin DarT in some bacteria species.^15^ Other tested NADAR domains, including those from eukaryotic species, exhibited the same specificity *in vitro*. NADAR domains are small, mostly α-helical domains with a central active site that features a key catalytic aspartate or glutamate residue.^15^

In summary, research has revealed a large diversity of ADP-ribosylation eraser enzymes, involving different structural scaffolds and catalytic mechanisms. One of the practical consequences of this diversity is the existence of different enzymes with preference for different types of linkages, such as those between distinct protein amino-acid residues and ADP-ribose, or between two ADP-ribose units in either linear or branched PAR chains. This broad range of eraser specificities—combined with mechanisms of recruitment to specific locations and macromolecules and possible mechanisms of activation or inhibition—will define the function of erasers as parts of cellular signaling systems.

## ADP-ribosylation readers

Although an ADP-ribosylation event can directly change the activity of the substrate protein, more commonly it exerts its effects by modulating intermolecular interactions. Many MAR- and/or PAR-binding proteins have been reported, and recent high-throughput studies have identified a large number of further potential ADP-ribosylation readers.^89^^,^^90^ Readers can have preference for either MAR or PAR and, in case of PAR, chains with particular length or branching^18^ (Figure 3D, left). In some cases, the binding has been attributed to specific domains or motifs, several of which are described below in more detail. Several readers (macrodomains and tryptophane-tryptophane-glutamate [WWE] domains) have been used to produce antibody-like tools for detecting ADP-ribosylation and for enriching ADP-ribose-containing peptides and proteins from complex mixtures.^5^^,^^91^

### Types of ADP-ribosylation readers and their specificity

#### Macrodomain

The most widely distributed and diverse domain that binds to ADP-ribose and NAD^+^ is the macrodomain (Figure 3D, middle). It was described above in the context of ADP-ribosylation hydrolases but, when catalytically inactive, it can serve as an ADP-ribosylation reader.^53^ In general, macrodomain-containing readers bind to MAR or terminal ADP-ribose units of PAR chains. One of the human macrodomain-containing proteins that binds PAR is a chromatin remodeler, ALC1.^92^ In ALC1, the macrodomain recruits the ATP-dependent remodeling activity to PARylated nucleosomes and also inhibits ATP hydrolysis (through a direct intramolecular interaction) until PAR binding occurs.^93^^,^^94^^,^^95^^,^^96^ The major difference between reader macrodomains and their eraser counterparts is that, in the first type, the ADP-ribose moiety is bound in a relaxed configuration and that activating residues are absent from the binding site surrounding the distal ribose.

#### WWE domain

The characteristic structure of the WWE domain (named after three conserved residues) comprises a core antiparallel β sheet and an α helix reminiscent of the ubiquitin fold (Figure 3D, right). The best-characterized WWE domain is the one found in the ubiquitin E3 ligase really interesting new gene (RING) finger protein 146 (RNF146), also known as IDUNA, in which binding to PAR chains allosterically stimulates ubiquitylation activity.^54^ The WWE domain of RNF146 was shown to recognize *iso*-ADP-ribose, an intermediate molecule that consists of segments from two adjacent ADP-ribose units of a PAR chain, making it a PAR-specific reader. However, the binding ability and specificity of WWE domains may vary. This is illustrated by tandem WWE domains present in PARP13, of which the first domain seems to lack functionality in ADP-ribose binding, whereas the second domain exhibits a preference for recognizing the terminal unit in a PAR chain.^97^^,^^98^

#### Other reader domains and motifs

Poly(ADP-ribose)-binding zinc-finger (PBZ) domains, comprising a C2H2 zinc finger and found mainly in DNA repair and checkpoint proteins, achieve their PAR chain specificity by interacting with adenine rings of two consecutive ADP-ribose units.^99^^,^^100^ Other structured domains reported as readers of protein ADP-ribosylation include OB-fold, FHA and BRCT, PIN, and RNA-recognition motif (RRM) domains^101^^,^^102^ but structures of their ADP-ribose-bound states are lacking to confirm and visualize the proposed interactions. Additionally, ADP-ribose binding has been attributed to short, unstructured motifs, typically highly positively charged, including the canonical PAR-binding motif (PBM).^103^

The diversity of ADP-ribosylation readers—arguably exceeding that of readers of any other protein PTM—allows for complex downstream responses to MAR and PAR signals, which remain largely unexplored in their mechanistic details. However, compelling evidence underscores the involvement of ADP-ribosylation in numerous biological functions.

## Biological functions of ADP-ribosylation in eukaryotes

**Figure 4:**
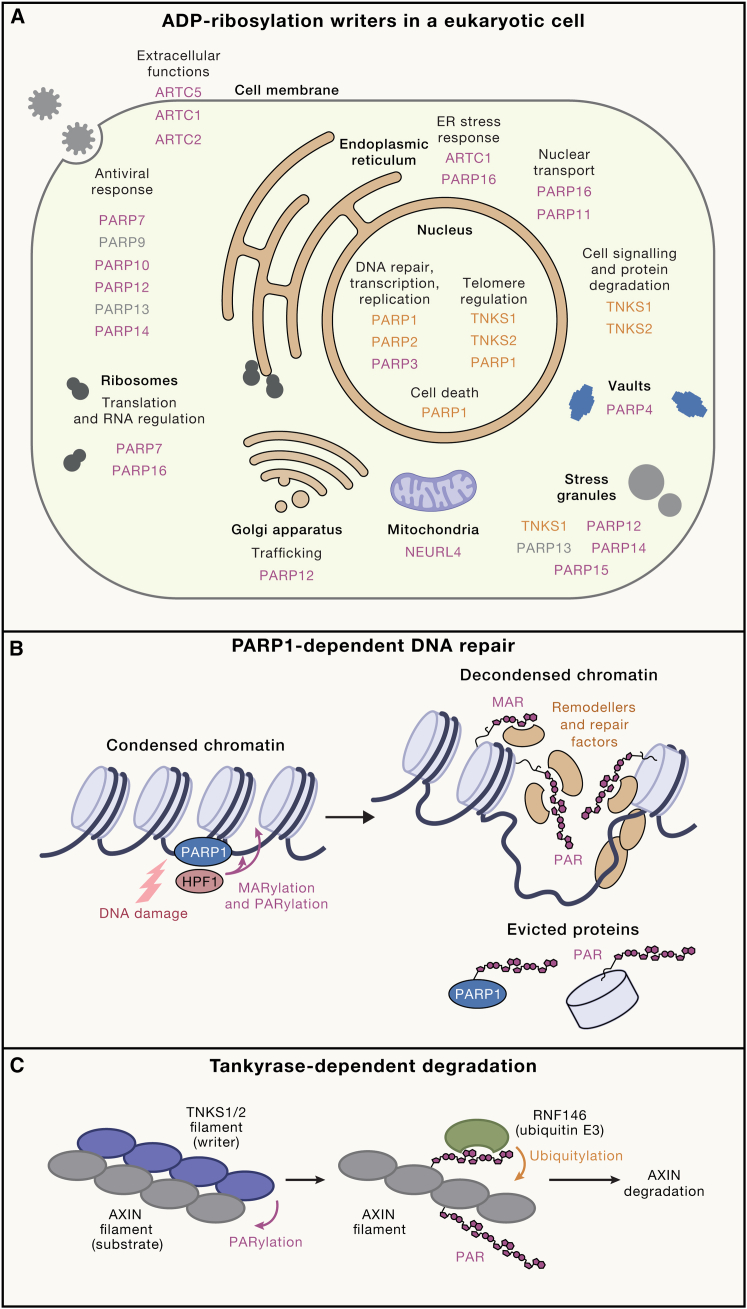
Molecular and cellular functions of ADP-ribosylation (A) Selective summary of localization and function of ADP-ribosylation writers in a eukaryotic cell. The figure does not provide a comprehensive list of known localizations, particularly for PARPs that have been detected in multiple cellular compartments. The enzymes capable of PARylation are shown in orange, the enzymes with MARylation but not PARylation activity are shown in purple, and enzymes for which no ADP-ribosylation activity has been identified are shown in gray. (B) Simplified scheme of PARP1-dependent regulation of DNA repair. PARP1 recognizes a DNA break, becomes activated, and catalyzes both MARylation and PARylation on PARP1 itself and other proteins, including histones. HPF1 is involved in the initial attachment of ADP-ribose to a protein but not in PAR chain elongation. Deposited MAR and PAR signals trigger chromatin decondensation, recruitment of chromatin remodelers and DNA repair factors, and dissociation of some proteins, including PARP1 itself and possibly histones or nucleosomes. (C) Implication of tankyrase-dependent degradation of proteins in cellular signaling illustrated using a representative substrate, AXIN. Tankyrases exist as noncovalent polymers (filaments), which might provide multivalency for recognizing polymeric substrates such as AXIN. Following tankyrase-mediated substrate PARylation, the PAR signals on the substrate are recognized by the PAR-directed ubiquitin E3 ligase RNF146, which catalyzes substrate ubiquitylation, triggering its subsequent proteasomal degradation.

ADP-ribosylation plays multiple roles in eukaryotes, related both to general maintenance and to the response to various types of stress and danger. In terms of more general pathways, ADP-ribosylation has been implicated in, among others, transcription, translation, RNA stability, spindle assembly and cell division, cell signaling, trafficking, and nuclear-cytoplasmic transport. As for pathways related to stress management, immunity, and defense, ADP-ribosylation has links to both innate and adaptive immunity (especially against viruses), protein quality control, and DNA damage response (DDR). These various functions have been reviewed in recent years.^5^^,^^76^^,^^104^^,^^105^ Below, some of them are covered, grouped by the specific associated ADP-ribosylation writers with a focus on human biology. Illustrative examples of functions of various writers in different cellular compartments are also shown in Figure 4. Although correct ADP-ribosylation responses rely on writers, erasers, readers, and substrates, we focus on writers, which play a defining, initiating role by producing the ADP-ribose signals.

### PARP1

PARP1, the paradigmatic ADP-ribosylating enzyme, is one of the most abundant nuclear proteins, with a nuclear concentration of about 20 μM.^71^ PARP1 plays a role in a plethora of cellular processes.^76^ PARP1’s best-characterized function is in DDR and DNA repair, where PARP1 is best known for detecting single-strand DNA breaks (SSBs) and initiating the recruitment of SSB repair factors via a scaffolding protein, XRCC1.^106^ PARP1 also regulates homologous recombination (HR) and non-homologous end joining (NHEJ), as well as base excision repair (BER) and nucleotide excision repair (NER).^76^

At the molecular level, PARP1 acts as a first responder that detects and becomes activated by different forms of DNA breaks.^48^^,^^76^ Once activated, PARP1 catalyzes ADP-ribosylation of itself and other protein substrates present in the vicinity of a lesion, in particular histones and other chromatin-associated proteins. This leads to chromatin decondensation and recruitment of DNA repair factors, many of which harbor MAR or PAR reader domains^107^ (Figure 4B). Chromatin relaxation is both a direct consequence of histone ADP-ribosylation and a result of the action of PAR-binding ATP-dependent nucleosome remodelers and histone chaperones, such as ALC1^92^ and APLF,^108^ with recent reports of PAR on specific histone serine sites transforming nucleosomes into robust ALC1 substrates.^95^^,^^109^ The initial rapid burst of PARP1-mediated PARylation appears to be followed by a slower second wave of PARP1-dependent MARylation, which differ in the type of recruited factors.^110^ Both types of DNA break-induced ADP-ribosylation are largely dependent on HPF1,^56^^,^^75^^,^^107^^,^^111^ which is necessary for the initiation of protein ADP-ribosylation on serine residues but not for PAR chain elongation.^17^ DNA damage-dependent ADP-ribosylation is reversed by the combined action of PARG and ARH3, both of which are recruited to DNA damage.^111^^,^^112^^,^^113^ PARG is the main hydrolase responsible for trimming of linear and branched PAR chains.^25^ By contrast, ARH3 is primarily responsible for cleaving the bond between the initial ADP-ribose and a serine residue,^114^ and, to some extent, contributes to linear but not branched PAR digestion^52^ and possible cleavage of initial ADP-ribose units linked to a glutamate residue.^115^

In addition to DNA breaks, PARP1 binds to and is activated at stalled replication forks, reducing replication fork speed to relieve replication stress. Consistently, inhibition of PARP1 increases replication fork speed, leading to genome instability.^116^ The exact mechanism underlying PARP1’s impact on replication forks remains unclear. The methyl transferase CARM1 has been found to stimulate PARP1 binding to ssDNA gaps, enabling PARP1:HPF1-mediated ADP-ribosylation at replication forks.^117^ This is consistent with PARP1’s ability to bind to incompletely processed Okazaki fragments that have escaped canonical FEN1- and LIG1-mediated fragment sealing, which leads to recruitment of XRCC1 to initiate the processing. As a result, PARP1 contributes to nascent DNA maturation during replication, particularly on the lagging strand.^118^^,^^119^

In addition to its role in DNA repair and replication, PARP1 regulates many other cellular processes, including, but not limited to, chromatin organization, regulation of other PTMs, gene expression, ribosome biogenesis, RNA processing (splicing, maturation, stability, and export), translation, biomolecular condensate formation, proteostasis, and cell death (recently reviewed^76^). The sites of serine-ADP-ribosylation often overlap with other modifications, including phosphorylation (on the same serine) and acetylation and methylation (on the neighboring lysine), resulting in a crosstalk with potential biological relevance.^120^^,^^121^ While the roles of PARP1 have been studied for over 50 years, less attention has been given to other PARP proteins, which have only recently emerged as key cellular factors responsible for various functions.

### PARP2 and PARP3

PARP1 is most closely related to PARP2 and PARP3, which, while lacking some of PARP1’s DNA-binding domains, are activated by DNA breaks in a similar manner to PARP1. PARP2 is partially functionally redundant with PARP1, as demonstrated through the embryonic lethality of the double *PARP1* and *PARP2* knockout mice,^122^ but in some DNA repair situations, it might come chronologically after PARP1 to enrich branched PAR.^123^ Although PARP2 is similar to PARP1 in its involvement in SSB repair, its dependence on HPF1, and its ability to catalyze PARylation, PARP3 does not interact with HPF1 and catalyzes only MARylation.^6^^,^^9^^,^^56^^,^^124^ PARP3 is stimulated by double-strand DNA breaks (DSBs) and predominantly contributes to DSB repair,^125^^,^^126^^,^^127^ but it can also bind to and initiate repair of SSBs and has a role in mitotic progression.^128^

### Tankyrases

TNKS1 and TNKS2 are the next most studied members of the family, with various cellular roles reported, including in WNT-β-catenin signaling, telomere regulation, membrane trafficking, and immunity.^129^ Mechanistically, the best-understood aspect of tankyrase signaling is the activation of the WNT-β-catenin pathway by binding to and PARylating its main negative regulator, AXIN, thus triggering ubiquitylation of the PARylated AXIN by the E3 ubiquitin ligase RNF146^130^ (Figure 4C). Tankyrase-mediated ADP-ribosylation has been shown to be involved in immunity and inflammation through the regulation of the tumor necrosis factor (TNF)-induced death.^131^ Moreover, tankyrases were identified as critical players in telomere integrity^132^ and in the stabilization of the mitotic spindle, notably by regulating the spindle-pole protein NuMA.^133^

### Antiviral PARPs

The expression of many MARylating PARPs, namely PARP7, PARP10, PARP11, PARP12, and PARP14, together with PARP9 and PARP13, which lack ADP-ribosylation activity, is stimulated by interferons or viral infection and constitute a part of the antiviral defense system.^134^^,^^135^^,^^136^^,^^137^ However, the molecular events underlying the immunity-related biological effects of these various PARPs remain unclear. PARP7, PARP9, PARP10, PARP12, PARP13, and PARP14 contain putative or confirmed RNA-binding domains, strongly suggesting their roles in RNA-related physiological processes and/or antiviral defense as RNA sensors.^29^^,^^138^ Some of the PARPs’ antiviral roles could involve RNA ADP-ribosylation, particularly at phosphorylated ends.^10^^,^^11^ Moreover, most of these PARPs also possess PAR-binding domains, highlighting their likely crosstalk with PARP1 or other family members.^29^ PARP14, in addition to possessing a writer ART domain and an ADP-ribose reader macrodomain, also harbors a catalytically active eraser macrodomain, whose role might be to fine-tune PARP14’s ADP-ribosylation output.^139^^,^^140^ Viruses appear to have appropriated the eraser macrodomain of an ancestral PARP14 protein to use it to counteract the host’s antiviral response.^140^ A special case of an “antiviral PARP” is represented by PARP9, which, while itself likely inactive as an ADP-ribosylation writer, makes a complex with DTX3L, a ubiquitin E3 ligase that can ubiquitylate both protein lysine residues and the ADP-ribose moiety.^19^ In the latter case, this can lead to the creation of composite ubiquitin-ADP-ribose signals on proteins or other substrates, which could play a role in immunity. Of note, PARP9 has been found to play a role in cellular defenses from viruses and *Mycobacterium tuberculosis*, interestingly, through opposing effects on interferon production: its induction in response to viral infections^134^ and its suppression in the case of *M. tuberculosis* infection,^141^ both of which are protective.

### Noncanonical PARPs

#### PARPs and nuclear events

PARP7, PARP9, PARP10, and PARP14 have been reported to localize to the nucleus under certain conditions and could contribute to transcription, cell cycle regulation, and genome stability maintenance, including replication fork stability, as recently reviewed.^142^^,^^143^ PARP10, in particular, has been implicated in regulating nuclear factor κB (NF-κB) signaling^144^ and cell cycle regulation.^145^^,^^146^^,^^147^ PARP11 is primarily located at nuclear pores and plays a role in maintaining nuclear envelope stability and remodeling by MARylating several nuclear pore complex proteins.^9^^,^^142^ Interestingly, inhibition of PARP11 results in its disassociation from the nuclear envelope.^148^ Future studies are needed to determine if catalytic activity is a more general regulatory mechanism of PARP localization.

#### PARPs in the ER, the Golgi, and stress granules

In line with its primarily ER membrane localization, PARP16 has been reported to be involved in the unfolded protein response by MARylating and activating key ER kinases, PERK and IRE1α,^34^ and to inhibit translation and maintain proteostasis in cancers by MARylation of ribosomal proteins.^7^ Another substrate of mammalian PARP16 is karyopherin, suggesting that PARP16 could be involved in nuclear-cytoplasmic transport.^35^ PARP12 is involved in membrane trafficking by MARylating Golgin-97^149^ and can translocate from the Golgi complex to stress granules in a reversible manner dependent on PARP1-mediated PARylation.^150^ Additionally, PARP12 and PARP13 were shown to contribute to the formation of stress granules, cytoplasmic condensates composed of proteins and nucleic acids.^98^^,^^150^ The repeatedly reported link between PARPs and cellular membraneless compartments including stress granules^30^^,^^150^^,^^151^^,^^152^^,^^153^^,^^154^ might be connected with the ability of ADP-ribosylation to trigger protein condensation, which has been observed *in vitro*. In one study, PAR was able to trigger condensation of FUS at very low concentrations (1 nM) and substoichometric amounts.^151^ Although PAR appears to be a particularly potent condensation inducer, it is currently unclear to what extent PARP1 or other PARylating PARPs cooperate with MARylating PARPs in establishing stress granules and other condensates.

#### Search for PARP substrates

Despite the recent progress in investigating functions and substrates of PARPs other than PARP1 or PARP2 or tankyrases, cellular targets of these PARPs have not been fully identified or validated and in some cases remain almost completely unknown. Arginine, tyrosine, histidine, and cysteine ADP-ribosylation sites, which have been detected as the most abundant after serine sites,^155^ could be specific to certain MARylating PARP enzymes, in addition to non-proteinaceous targets such as nucleic acids. Future research, particularly unbiased proteomics analyses, will undoubtedly clarify the substrate preferences of MARylating PARPs, with recent studies already highlighting dual specificity of PARP7 for cysteine and aspartate/glutamate residues and its role in the functional regulation of the proteins involved in cytoskeleton organization, nuclear receptor signaling, RNA metabolism, and innate immunity.^156^^,^^157^^,^^158^^,^^159^

### ARTC enzymes

Of the five mammalian ARTC proteins, ARTC2, studied in mice,^160^ is not produced in humans due to a premature stop codon, ARTC3 and ARTC4 are thought to be catalytically inactive, and ARTC5 is a secreted enzyme that might be active predominantly as an NAD^+^ hydrolase.^36^ ARTC1, therefore, remains as the major ADP-ribosylating ARTC in human cells. With its luminal ER localization and an arginine-specific protein MARylation activity, ARTC1 might account for the enrichment of arginine-linked ADP-ribosylation reported in the ER.^121^^,^^155^^,^^161^ Recent studies have reported that ARTC1 contributes to ER stress response and promotes immune resistance and other cancer hallmarks in cancer cells.^36^^,^^162^^,^^163^

In summary, eukaryotic PARPs and ARTC enzymes are found in virtually all parts of the cell and participate in multiple cellular pathways. Although most attention has been given to their role in the regulation of nuclear processes, especially that of PARP1 in DNA repair, recent years have expanded our understanding of the roles of ADP-ribosylation in other functions, including cellular signaling, RNA processing, and the antiviral response. Notably, understanding of ADP-ribosylation has been expanded by considering its role in bacteria and viruses.

## Bacterial ADP-ribosylation

Bacterial ADP-ribosylation was first discovered as a warfare mechanism, as in the case of diphtheria toxin and related bacterial exotoxins. However, ADP-ribosylation appears to be widespread in bacterial species with emerging evidence of being tightly controlled by dedicated writers and erasers, and serving regulatory roles, including regulation of growth and metabolism.^21^^,^^46^^,^^164^

### Reversible ADP-ribosylation-based regulation

The first characterized reversible protein ADP-ribosylation system in bacteria was the DraTG system, which regulates nitrogen fixation through arginine-linked ADP-ribosylation in several nitrogen-fixing proteobacteria.^164^ Another example is provided by reversible addition/removal of ADP-ribose to the carrier protein GcvH-L catalyzed by a class of sirtuins as writers and a macrodomain as an eraser.^42^ This system is found in bacterial pathogens including *Staphylococcus aureus* and *Streptococcus pyogenes* and has been linked to resistance to an oxidative immune response of a host. Interestingly, the ADP-ribosylation modification in this system is dependent on prior substrate lipoylation, with the crosstalk between these two PTMs being important for the response of pathogens to oxidative stress. Overall, reversible ADP-ribosylation systems appear to be a potent mechanism through which bacteria respond to the environment.

### DNA base ADP-ribosylation and DarTG toxin-antitoxin systems

An interesting aspect of bacterial ADP-ribosylation is the use of DNA bases as an acceptor. The first example of DNA ADP-ribosylation in bacteria has been provided by pierisin-like toxins of the ARTC family used by some *Streptomyces* and *Bacillus* species, among others, for irreversible modification of host DNA.^21^ These toxins are related to the irreversible toxins described in butterflies, such as pierisin-1.^165^ An example of reversible DNA ADP-ribosylation is provided by toxin-antitoxin systems composed of a PARP-like ARTD writer DarT and an eraser DarG, where the latter component is either a macrodomain- or a NADAR domain-containing hydrolase (DarG2 or DarG1, respectively).^14^^,^^15^ The longer-known and better characterized of the two is the system composed of DarT and DarG2, which is found in various species, including *M. tuberculosis*. DarT-DarG2 acts through sequence-specific addition/removal of ADP-ribosylation to the thymine base in ssDNA, with the resultant DNA-ADP-ribosylation products perceived as DNA damage.^14^^,^^46^ Physiologically, DarT from this system targets replication origins and slows down growth of *M. tuberculosis*, potentially resulting in persistent infection and antibiotic tolerance,^46^ as well as providing phage defense in proteobacteria by modifying phage DNA, blocking viral DNA and RNA synthesis, and leading to abortive infection.^166^ The macrodomain enzyme DarG2 is a homolog of human TARG1^87^ and specifically reverses DarT-mediated DNA-ADP-ribosylation, restraining excessive toxicity, which makes DarG2 an essential component and a potential drug target for tuberculosis and other diseases caused by pathogens that express this system.^14^ The other DarTG system, featuring the DarG1 hydrolase, has been reported more recently in various bacteria, including some *Escherichia coli* strains.^15^^,^^166^ Although the DarT present in this system is related to that from DarG2-DarT, it exhibits altered specificity, ADP-ribosylating the guanine rather than thymine base in ssDNA.^15^ This change coincides with replacement of DarG2 by NADAR-containing DarG1 as a cognate antitoxin that specifically removes ADP-ribosylation from guanine in ssDNA.

### ADP-ribosylation-mediated noncanonical ubiquitylation

A mechanism recently discovered in the intracellular bacterial pathogen *Legionella pneumophila* gives a new twist to the old concept of host-targeting ADP-ribosylating toxins. This species, which is a causative agent of the Legionnaires’ disease, utilizes arginine ADP-ribosylation to achieve noncanonical ubiquitylation of host proteins and derails host ubiquitylation signaling.^167^^,^^168^ In this pathway, ubiquitin is first ADP-ribosylated on a specific arginine residue, prior to the cleavage of ADP-ribose at the pyrophosphate moiety and the attachment of ubiquitin via the phosphoribose remnant to a serine residue on a host substrate protein, effectively creating a noncanonical ubiquitin-substrate linkage. Although this system appears to be limited to *Legionella*, it is interesting to ask if similar chemistry, involving cleavage of a substrate-linked ADP-ribose and concomitant attachment of a new element through the phosphoribose remnant, could be discovered elsewhere in biology.

### Other bacterial ADP-ribosylation systems

The diversity of ADP-ribosylation targets in bacteria is further broadened by the antibiotic-modifying ART Arr from *M. smegmatis*, which inactivates antibiotic rifamycin by ADP-ribosylating its hydroxyl group.^169^ A recently discovered toxin, RhsP2, ADP-ribosylates 2′ hydroxyl groups of double-stranded RNA (dsRNA), including many ncRNAs and the entire tRNA pool.^170^ Aberrant ADP-ribosylation of RNA species affects multiple cellular processes, including tRNA charging by aminoacyl-tRNA synthetases and ribonuclease P-mediated processing of polycistronic tRNAs, eventually leading to cell death. Finally, a few divergent ART domains have been discovered as a toxic part of Rhs polymorphic toxins that can be neutralized by their cognate immunity proteins either by protein:protein interactions or enzymatic removal of ADP-ribosylation. Tre1 (type VI secretion ART effector 1) in gram-negative bacteria acts as a toxic effector for intermicrobial competition by MARylating FtsZ, the prokaryotic tubulin homolog important for cell division. Cells employing this system might use an ARH-type ADP-ribosyl eraser as an immunity protein that reverses ADP-ribosylation of its own FtsZ.

The above examples show that bacteria have emerged in recent years as a rich reservoir of diverse ADP-ribosylation systems, which are largely unexplored. Further research into bacterial ADP-ribosylation holds significant promise, not only for understanding bacterial physiology and virulence but also for inspiring new hypotheses and developing novel tools to study eukaryotic systems.

## Viral ADP-ribosylation

ADP-ribosylation-related proteins have also been discovered in viruses, some of which—including notorious human pathogens—appear to use ADP-ribosylation writers or erasers to modulate the host’s ADP-ribosylation signaling. Although viral ADP-ribosylation is generally less understood than eukaryotic or bacterial, genomic data indicate involvement of this modification in virus-host interactions.^135^

### Viral ADP-ribosylation writers

Although ARTs are found only sporadically in phages and viruses, they have established roles in some of them.^21^ For example, T4 phages encode at least three ARTC-like ARTs that target various bacterial proteins, including RNA polymerase.^21^^,^^171^ Putative functional PARP-like proteins of unknown function have been identified in the *Aeromonas* phage Aeh1, nucleopolyhedroviruses, and invertebrate iridescent virus 6.^26^

### Viral ADP-ribosylation erasers

In contrast to ADP-ribosylation writers, eraser domains are found in many viral multidomain proteins, with macrodomains being the most prevalent.^26^ The macrodomain Mac1, a MacroD-type macrodomain which is a part of the NSP3 protein of coronaviruses, including severe acute respiratory syndrome coronavirus 2 (SARS-CoV-2), counteracts the activity of antiviral PARPs and is essential for the replication of the coronavirus *in vivo*.^172^^,^^173^ Homologs of Mac1/MacroD-type domains are also found in alphaviruses, Hepatitis E, and Rubella. The apparent arms race between host’s PARP enzymes such as PARP14 and viral macrodomain hydrolases represents a fascinating example of an ADP-ribosylation-based mechanism that holds therapeutic potential for tackling emerging infectious diseases.^137^^,^^140^^,^^172^

## ADP-ribosylation in human disease and therapy

In addition to ADP-ribosylation systems implicated in bacterial or viral infections, the primary connection between ADP-ribosylation and human health comes from sensitivity of some cancer phenotypes to the inhibition of PARP1 and PARP2. Below, we review the current and potential clinical applications of PARPi, including recent developments such as a PARP1-specific inhibitor, inhibitors of other PARPs, and applications of PARPi to non-oncological indications. It should be born in mind that in addition to their clinical potential, PARPi are also potent tools for research into human ADP-ribosylation.

### PARP inhibitors in cancer therapies

#### PARP1 and PARP2 inhibitors

The term PARPi is typically used for molecules that are primarily PARP1- and PARP2-specific, although most of them have a nonnegligible effect on some of the other PARPs.^174^ Most known PARPi block the PARP catalytic ADP-ribosylation activity by using aromatic groups to occupy the deep NAM-binding pocket and outcompete NAD^+^.^175^^,^^176^ Indeed, it could be argued that the presence of this pocket accounts for PARPs’ ready druggability, leading to very high affinity and slow dissociation observed for some PARPi. Four PARPi, namely olaparib, rucaparib, niraparib, and talazoparib, have been approved for the treatment of breast, ovarian, pancreatic, or prostate cancers, primarily those with defects in HR genes *BRCA1* or *BRCA2*, with which PARP1 or PARP2 inhibition is synthetic lethal.^3^^,^^4^ With a view to reducing toxic side effects of PARPi that seem to be caused primarily by PARP2 inhibition, more selective PARP1 inhibitors such as AZD5305 have been developed^177^ and entered clinical trials. In addition to the established use of PARPi against *BRCA*-deficient cancers, numerous preclinical and clinical studies are currently aimed at extending the oncological use of PARPi to other cancers, whether as monotherapy or in combination with other drugs. These include cancers with defects in other HR genes, such as *PALB2*, *RAD51B*, or *RAD51C*. However, as these mutations are rare, genes from other pathways, e.g., *XRCC3*, *RNASEH2B*, *ALC1*, *DNPH1*, and *HPF1*, are being considered as better predictors of PARPi response, alongside phenotypic characterization.^75^^,^^178^

#### Mechanisms of action of PARPi and PARP trapping

PARPi impair processes that involve PARP1 or PARP2, such as the repair of SSBs (leading to the production of DSBs, which could explain synthetic lethality with HR defects^76^) and the maturation of nascent DNA during replication (causing the accumulation of lagging strand ssDNA gaps^118^). These effects might, at least in part, depend on noncovalent trapping of PARP1 and PARP2 on damaged chromatin, which is observed upon PARP inhibition,^77^ although the nature, prevalence, and importance of PARP trapping are currently subjects of debate. Trapped PARP1 or PARP2, unlike merely inhibited forms of these PARPs, are thought to not only fail to facilitate but also actively interfere with, DNA repair and other nuclear transactions.^76^^,^^77^ Recent research suggests that, in general, trapping is not equivalent to physical stalling of individual PARP molecules on DNA and may mainly result from continuous PARP rebinding.^67^ PARP rebinding appears to be caused primarily by the inhibition of PARP automodification, which normally serves to terminate PARP accumulation at damage sites. Actual stalling of PARP molecules—which might be advantageous from a therapeutic perspective—has been observed with compounds that allosterically enhance the PARP:DNA interaction.^66^ In PARP1, such an effect has so far been reported only with nonclinical inhibitors.^66^^,^^179^ In PARP2, more inhibitors, including clinically relevant PARPi, have been shown to lead to allosterically induced stalling due to subtle structural differences from PARP1.^180^^,^^181^

#### PARPi resistance

Although PARPi have lasting positive effects in some patients, innate and acquired resistance is frequent and leads to disease relapse, calling for new therapy improvement strategies. Multiple genomic screens have been employed to identify potential mechanisms of PARPi resistance and ways of overcoming it.^182^^,^^183^ Briefly, the proposed resistance mechanisms (recently reviewed^175^^,^^183^) include (1) reversion mutations in *BRCA1*, *BRCA2*, and other HR genes; (2) non-reversion HR restoration, e.g., through the loss of 53BP1 or its downstream factors; (3) restoration of replication fork stability, e.g., upon EZH2 depletion, impaired recruitment of MRE11 nuclease, or loss of SLFN11; (4) *PARP1* point mutations; (5) restoration of ADP-ribosylation signaling, e.g., via inactivation of PARG or ARH3 hydrolases; (6) removal of trapped PARP1, e.g., through the activity of VCP/p97 or ALC1; and (7) upregulation of PARPi efflux. Among these mechanisms, only reactivation mutations or promoter hypermethylation of *BRCA1* and reversion mutations of *BRCA2*, *PALB2*, *RAD51C*, and *RAD51D* have been clinically validated,^183^ while others are known only from single clinical cases or preclinical data. PARPi resistance could be tackled by combining these drugs with agents that target the resistance mechanism(s), for example, checkpoint inhibitors to overcome restored replication fork stability.^184^

#### Emerging strategies of inhibiting noncanonical PARPs

The clinical success of inhibitors of PARP1 or PARP2 has fueled interest in exploring the therapeutic benefits of inhibiting other PARPs. Inhibitors of tankyrases (the selective inhibitor STP1002 and the dual PARP1/PARP2 and TNKS1/TNKS2 inhibitor E7449), PARP7 (RBN-2397), and PARP14 (RBN-31430) are currently undergoing clinical trials for treatment of cancer and inflammatory diseases.^157^^,^^185^^,^^186^ Further research will help to identify additional therapeutic opportunities. For instance, a recent study suggested PARP14 inhibition as a promising approach in the treatment of follicular lymphomas with *STAT6* mutations that lead to increased *PARP14* expression.^187^ In addition to PARP7 and PARP14, other MARylation enzymes, including PARP3, PARP8, PARP10, PARP11,^142^ as well as an ARTC family member, ARTC1,^162^ have been proposed as potential targets in cancer therapies. For instance, the selective PARP11 inhibitor ITK7^148^ could target PARP11-induced immunosuppression in tumors.^188^

### Therapeutic potential of PARP inhibitors in non-oncological pathologies

PARPi not only allow for exploiting cancer vulnerabilities but also hold potential in treating non-oncological conditions by counteracting excessive ADP-ribosylation that may cause or aggravate some non-cancerous conditions.^175^ Excessive ADP-ribosylation, either through overactivation of PARP1 (and possibly other PARPs) or due to the loss of ADP-ribosylation erasers,^87^^,^^189^ has been implicated in a range of human pathologies, including different forms of neurodegeneration, immune and inflammatory diseases, cardiovascular conditions, and various injuries.^175^ Toxic consequences of increased ADP-ribosylation might be due to NAD^+^ or ATP depletion or the accumulation of ADP-ribosylation signals themselves, especially PAR chains.^17^ In the latter case, several mechanisms have been proposed to explain PAR’s harmful effects, including cell death induction, promotion of protein aggregation, and stimulation of proinflammatory signaling.^175^^,^^190^ Although the results of preclinical studies in this area, generally involving repurposing of anti-cancer PARPi, have been promising,^175^ further research and clinical trials are needed to determine safety and effectiveness in humans. This is particularly the case with respect to central nervous system diseases, where the blood-brain barrier poses a challenge for drug delivery.

In summary, although ADP-ribosylation has already proven its high medical relevance in cancer through the success of canonical PARPi, the field is currently exploring new possibilities, such as developing more specific PARP1 inhibitors, therapies to address PARPi resistance, combination treatments, inhibitors that target other PARPs, and investigating the potential applications of PARPi for non-cancer-related conditions.

## Concluding remarks

Following rapid advances in the ADP-ribosylation field over the last several years, we are currently at the point where, in addition to many fundamental questions investigated for decades (for which we still have only partial answers), we now face also new, unexpected questions prompted by recent discoveries. This situation reveals the complexity of the ADP-ribosylation system and even more increases the desire for further knowledge.

To begin with, we are still far from having a clear picture of the catalytic mechanisms of various ADP-ribosylation writers and erasers, especially those that lack residues that play catalytic roles in model enzymes, e.g., PARPs lacking the catalytic glutamate. The situation is more complicated if we consider that some enzymes might utilize accessory factors, as recently discovered in the case of PARP1 and PARP2, which interact with HPF1. Except for a few best-studied examples, we know even less regarding how writer and eraser enzymes select their specific substrates and sites and how they achieve specific linkage chemistry. Moreover, in many cases, we are actually still not certain which substrates, sites, and linkage types are preferred by a given enzyme under physiological conditions. Similarly, exact specificities of various reported ADP-ribosylation readers remain to be fully elucidated. These questions are multiplying as the field is moving from focusing on a few model proteins to a more comprehensive study of ADP-ribosylation writers, erasers, and readers.

Connected with mechanistic properties of individual enzymes and readers is the question of mutual independence—or perhaps some functional interplay—of ADP-ribosylation signals linked to proteins via different amino acids. For example, serine- and glutamate-linked ADP-ribosylation could, in principle, be produced and removed by different enzymes (or different complexes, as in the case of PARP1 alone and PARP1:HPF1) and, perhaps, recognized by different readers. However, do these signals really function independently in cells? Similarly, to what extent is ADP-ribosylation signaling in various cellular compartments mutually independent? Such questions are further complicated by the possible interplay between MARylation and PARylation. Could PARP1/PARP2 or tankyrases extend chains initiated by other, MARylating PARPs or non-PARP ARTs?

As PARylation can have a particular length and be linear or branched, we face a further question of whether these structural differences within PAR might translate into different functional outcomes, leading to the idea of the PAR code. Attractive as this concept is, it still awaits more detailed validation. Moreover, the question of signal complexity is made more difficult if MAR or PAR signals can be covalently coupled with other PTMs, as in the case of ubiquitylation of ADP-ribose linked to proteins, which has recently been observed *in vitro*. Are these and perhaps other modified ADP-ribosylation signals found in cells, and if yes, do they have dedicated functions, e.g., recruitment of specific readers? Modified ADP-ribosylation signals provide a twist on a more familiar theme, of interplay between ADP-ribosylation and other PTMs not through a direct covalent link but on the same protein substrate, which also needs further exploration.

In addition to a global picture of different ADP-ribosylation signals, we ultimately need to uncover specific mechanisms, linking, step by step, (de)modification events on particular substrates with defined biological outcomes. The number of cases where such mechanisms have been elucidated is growing but is still small. If we consider that not only proteins, but likely also non-proteinaceous substrates such as nucleic acids might be ADP-ribosylated in large numbers in cells, there are potentially very many distinct regulatory events waiting to be characterized. Finally, these questions get more complex as we shift from *in vitro* systems and model cell lines to a higher, organismal level. From a medical point of view, one ADP-ribosylation-related process whose elucidation might be particularly timely is the interaction between antiviral human PARPs and ADP-ribosyl erasers present in viruses, including SARS-CoV-2.

Another fundamental mechanism that has captured considerable attention in recent years is PAR’s ability to trigger macromolecular condensates at surprisingly low concentrations *in vitro*. Although there is accumulating indirect evidence associating ADP-ribosylation with membraneless compartments in cells, work still needs to be done to translate *in vitro* models to phenomena that happen in a complex cellular milieu. We expect exciting findings in this area in the coming years.

From the point of view of human therapy, the focus over the last decade has predominantly been on two uses for PARPi: first and foremost, targeting genome stability defects in cancer, and second, protecting non-cancerous cells from excessive ADP-ribosylation in some non-oncological conditions. However, we are already seeing, and will surely continue to see, the development of new concepts related to the inhibition of PARPs other than canonical PARP1 and PARP2 as well as ADP-ribosylation erasers and probably readers. The use of targeted protein degradation and various forms of combination therapy could further improve therapeutic prospects.

60 years after its inauguration, the ADP-ribosylation field might just be on the brink of yet another major breakthrough.
